# Endobronchial One-Way Valve Therapy Facilitates Weaning from Extracorporeal Membrane Oxygenation in a Patient with ARDS and Persistent Air Leak

**DOI:** 10.1155/2018/9736217

**Published:** 2018-09-25

**Authors:** Alessandro Ghiani, Matthias Hansen, Konstantinos Tsitouras, Claus Neurohr

**Affiliations:** ^1^Schillerhoehe Lung Clinic (Robert-Bosch Hospital), Department of Pneumology and Critical Care Medicine, Solitudestr. 18, 70839 Gerlingen, Germany; ^2^Schillerhoehe Lung Clinic (Robert-Bosch Hospital), Department of Anesthesiology and Critical Care Medicine, Solitudestr. 18, 70839 Gerlingen, Germany

## Abstract

Prolonged pulmonary air leak (PAL) is a common clinical problem, associated with significant morbidity and mortality. There are numerous reports of treatment of PAL using endobronchial valves (EBV) in respiratory stable patients, but only few reports on critically ill patients, and there is virtually no practical knowledge in the treatment of PAL in mechanically ventilated patients with acute respiratory distress syndrome (ARDS), treated with veno-venous extracorporeal membrane oxygenation (vvECMO). We describe a case where EBV placement was performed in a patient with ARDS and PAL, treated with mechanical ventilation and vvECMO. Despite a lung protective ventilation strategy, a persistent air leak along with a large left-sided pneumothorax was observed. After bronchoscopic localisation of the fistula, two endobronchial valves were inserted into the left upper lobe, leading to an immediate decrease in the air flow and reexpansion of the left lung. During the following two weeks, the patient was weaned from vvECMO, and after another three weeks, complete liberation from mechanical ventilation was accomplished. EBV placement seems to be a safe method even in the presence of coagulopathy and may facilitate mechanical ventilation and weaning from vvECMO in patients with ARDS and PAL.

## 1. Introduction

Prolonged pulmonary air leaks (PAL) are common and may cause considerable morbidity, prolonged hospital stay, and increased health-care costs [1]. They occur in about 15% of patients after thoracic surgery [2] or develop spontaneously due to an underlying pulmonary disease such as bullous emphysema, advanced interstitial lung disease, lung cancer, or cavernous tuberculosis. Poor performance status caused by pulmonary disease may limit surgical interventions. Therefore, less invasive therapeutic options have been developed in the past 20 years, such as fibrin sealants [3], metal coils [4], chemical pleurodesis [5], and endobronchial valves (EBV) [6].

There is little practical knowledge in the treatment of PAL in mechanically ventilated patients with acute respiratory distress syndrome (ARDS) [7] and even less experience in patients with ARDS and PAL, treated with veno-venous extracorporeal membrane oxygenation (vvECMO) [8].

We describe a case where bedside EBV placement was performed in a patient with ARDS and PAL, while being on invasive mechanical ventilation and vvECMO.

## 2. Case Report

**Figure 1:**
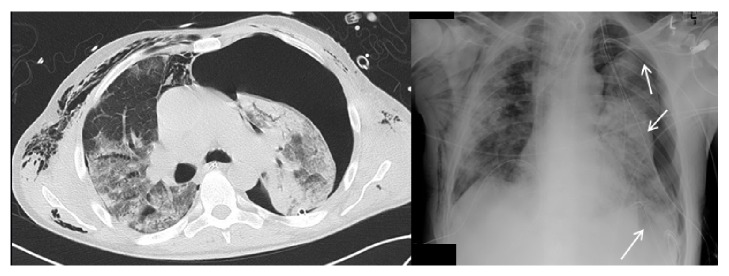
CT-scan (left image) and chest X-ray (right image) on day 16 before endobronchial valve placement: large pneumothorax despite three chest drainages on the left (white arrows).

A 60-year-old healthy male patient (no comorbidities, never-smoker) with ARDS due to influenza A pneumonia was admitted to our specialised lung clinic for further treatment. Endotracheal intubation due to severe hypoxemic respiratory failure was already performed prior to admission (day 0). Transference of the patient to the hospital occurred on day 6. The first chest X-ray after admission revealed an apical left-sided pneumothorax of approximately 2 cm, accompanied by a large subcutaneous emphysema. Two chest tubes were inserted, one on each side. After that, the left lung was again fully expanded, the subcutaneous emphysema resolved, and no air leak could be observed. On day 14, the patient developed once again spontaneously a massive and progressive subcutaneous emphysema, accompanied by a large air leak of about 5000 ml per minute on the left side. A second and a third chest tube were inserted on the left, one in Monaldi's position (3rd intercostal space mid-clavicular) and the other in Bülau's position (5th intercostal space slightly anterior the mid-axillary line). However, the chest X-ray and chest CT-scan on day 16 showed a complete, left-sided pneumothorax (Figure 1). Central venous catheter placement on the left (internal jugular vein) was performed six days before the onset of the air leak. We interpreted the occurrence of the air leak, the pneumothorax, and the subcutaneous emphysema as a result of barotrauma due to invasive mechanical ventilation. Because of progressive hypercapnia and severe respiratory acidosis, a tracheostomy was performed and a vvECMO (PLS Set and ROTAFLOW Console) was established (day 16), using an Avalon Elite™ Bi-Caval Dual Lumen Catheter (Maquet Cardiopulmonary GmbH, Germany). Blood gas analysis immediately before the start of the extracorporeal lung assistance revealed a P_a_CO_2_ of 88 mmHg with a pH of 7.30, and P_a_O_2_ was 90 mmHg. At this point, the patient was ventilated in the assist-controlled mode (BIPAP-ASB, Evita 4, Dräger®). F_i_O_2_ was 0.85, and P_insp_ was set at 28 mBar with a PEEP of 7 mBar. This resulted in a tidal volume of approximately 400 ml and a minute ventilation of 8.4 l/min. After the cannulation of the patient and the commencement of vvECMO support, a lung protective ventilation strategy with low tidal volumes (P_insp_ 20 mBar, PEEP 10 mBar, F_i_O_2_ 0.6, V_T_ 250 ml) was established. This resulted in acceptable blood gas values (P_a_CO_2_ 49 mmHg, P_a_O_2_ 74 mmHg, pH 7.52) on vvECMO (blood flow 2.5 l/min, F_i_O_2-vvECMO_ 1.0, sweep gas flow 4.0 l/min); however, the air leak persisted (day 17), and in the chest X-rays the left lung remained collapsed. In the further course of treatment, the blood gas analyses revealed persistent hypercapnia with a P_a_CO_2_ of approximately 60–65 mmHg, and even though the sweep gas flow was increased, a state of normocapnia could not be achieved. Therefore, an interventional closure of the fistula with endobronchial valves was planned.

**Figure 2:**
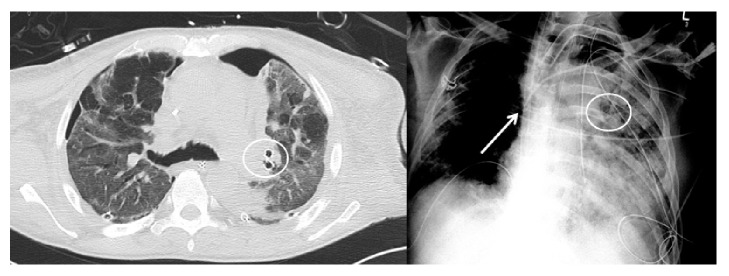
CT-scan (left image) and chest X-ray (right image) after endobronchial valve placement. The left lung is again fully expanded. The circled areas depict the two endobronchial valves within the left upper lobe segmental bronchi (LB1/2 and LB3). The Avalon Elite™ Bi-Caval Dual Lumen Catheter is visible on the chest X-ray (white arrow).

**Figure 3:**
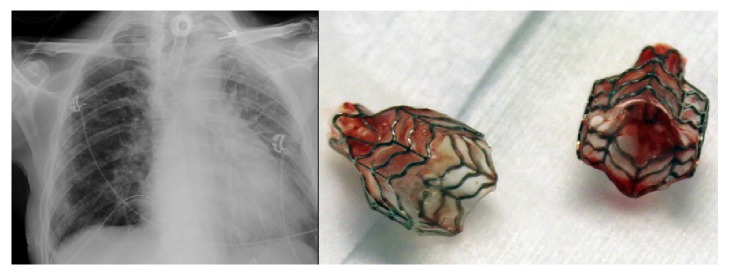
Chest X-ray (left image) after removal of the endobronchial valves (right image).

On the day of the procedure (day 21), F_i_O_2_ on the ventilator was 0.6, and P_insp_ was set at 17 mBar and PEEP at 5 mBar, resulting in tidal volumes of about 450 ml and minute ventilation of approximately 9.2 l/min. vvECMO blood flow was 2.6 l/min with a sweep gas flow of 5.5 l/min. Valve placement was done bedside on the intensive care unit. The patient was under deep sedation/analgesia with midazolam and sufentanil, respectively. Cis-Atracurium (10 mg) for muscle relaxation was administered immediately before the procedure. Bronchoscopy was performed through the tracheal cannula. The exact bronchoscopic localisation of the fistula was assessed by occlusion of the upper and lower lobe bronchus on the left, using a bronchus blocker while measuring the fistula flow with the Thopaz Digital Chest Drainage System (Medela AG, Switzerland). First of all, as we blocked the left upper lobe bronchus (corresponding to the segment bronchi LB1–5), the air leak was stopped entirely. Then we occluded each segment of the left upper lobe separately, but we did not achieve any significant result. The occlusion of the lingula bronchus (LB4/5) had no significant effect on the air leak as well. Thus, decision was made to close LB1/2 and LB3 with two Zephyr® endobronchial valves (2 × 4.0-LP, Pulmonx®, Redwood City, USA). This initially led to an immediate decrease of the air leak to about 400–700 ml/min, and the left lung was then again fully expanded (Figure 2). After valve placement, P_a_CO_2_ decreased slowly during the following six days, while there were no major adjustments of the ventilator settings or the vvECMO parameters. Normocapnia was detected for the first time on day 27, so that blood flow and sweep gas flow on vvECMO could be slowly reduced. During the following two weeks, the air leak stopped completely and the patient could be weaned from vvECMO on day 48. The patient was transferred from the intensive care unit to the weaning unit on day 61. Removal of the endobronchial valves occurred on day 62, after which the chest X-rays showed a persistently expanded left lung (Figure 3). As there was no evidence of an air leak once again, the chest tubes were removed one after another, liberation from the ventilator on day 72 and discharge to neurological rehabilitation on day 89.

## 3. Discussion

Persistent pulmonary air leak is a common clinical problem, associated with significant morbidity and mortality [9]. Large air leaks themselves can lead to respiratory failure, which may necessitate mechanical ventilation or even extracorporeal lung assistance. To date, only a few reports exist on mechanically ventilated, critically ill patients with PAL [10], and there is little practical knowledge in the treatment of PAL in patients with ARDS. Recently, a case series described endobronchial one-way valve placement as a feasible procedure in those patients [7].

In mechanical ventilation due to ARDS, high inspiratory and expiratory airway pressures may delay or even prevent the spontaneous closure of the fistula, because the persistent air flow through this low-resistance pathway averts healing of the affected lung. This effect is even enhanced by spontaneous breathing efforts during controlled mechanical ventilation, referred to as patient-ventilator asynchrony, which may generate highly negative pleural pressure swings and therefore increase the pressure gradient between the airway and the pleural cavity. This is why patients with ARDS have a low likelihood of unprompted resolution of large fistulas, as long as they are mechanically ventilated. We interpreted the observed residual air leak of 400–700 ml/min after placement of the two valves as “intralobar” collateral ventilation between the closed segments 1–3 (localisation of the fistula) and the ventilated segments 4/5 of the left upper lobe. In order to maintain gas exchange through the lingula segments, we decided to leave the lingula bronchus open, because in our experience such a low flow usually stops spontaneously during the following days.

To our knowledge, this is the second report on a patient with EBV treatment while being on vvECMO [8]. Under these circumstances, the risk of bleeding due to heparin anticoagulation is difficult to predict but must be weighed against the expected therapeutic effect. Nevertheless, the longer the air leak persists, the greater the likelihood of complications in the course of treatment will be, such as severe bleeding. Early bronchoscopic intervention in patients with PAL may shorten the duration of extracorporeal lung assistance and mechanical ventilation and, in our opinion, can help to avoid complications due to vvECMO.

## 4. Conclusion

Endobronchial valve placement seems to be a feasible procedure even in the presence of coagulopathy and may facilitate mechanical ventilation and weaning from vvECMO in patients with ARDS and PAL.
